# From stress fiber to focal adhesion: a role of actin crosslinkers in force transmission

**DOI:** 10.3389/fcell.2024.1444827

**Published:** 2024-08-13

**Authors:** Hiroki Katsuta, Masahiro Sokabe, Hiroaki Hirata

**Affiliations:** ^1^ Department of Cardiovascular Physiology, Graduate School of Medicine, Dentistry and Pharmaceutical Sciences, Okayama University, Okayama, Japan; ^2^ Human Information Systems Laboratories, Kanazawa Institute of Technology, Hakusan, Japan; ^3^ Department of Applied Bioscience, Kanazawa Institute of Technology, Hakusan, Japan

**Keywords:** stress fiber, focal adhesion, viscoelasticity, crosslinking protein, rigidity sensing, extracellular matrix, cell migration, mechanotransduction

## Abstract

The contractile apparatus, stress fiber (SF), is connected to the cell adhesion machinery, focal adhesion (FA), at the termini of SF. The SF-FA complex is essential for various mechanical activities of cells, including cell adhesion to the extracellular matrix (ECM), ECM rigidity sensing, and cell migration. This mini-review highlights the importance of SF mechanics in these cellular activities. Actin-crosslinking proteins solidify SFs by attenuating myosin-driven flows of actin and myosin filaments within the SF. In the solidified SFs, viscous slippage between actin filaments in SFs and between the filaments and the surrounding cytosol is reduced, leading to efficient transmission of myosin-generated contractile force along the SFs. Hence, SF solidification via actin crosslinking ensures exertion of a large force to FAs, enabling FA maturation, ECM rigidity sensing and cell migration. We further discuss intracellular mechanisms for tuning crosslinker-modulated SF mechanics and the potential relationship between the aberrance of SF mechanics and pathology including cancer.

## Introduction

The interaction between the stress fiber (SF) and the focal adhesion (FA) is crucial for numerous cellular processes, ranging from cellular structural maintenance and adhesion to the extracellular matrix (ECM) to ECM rigidity sensing, cell motility, and differentiation (Totsukawa et al., 1999; Kobayashi and Sokabe, 2010; Plotnikov et al., 2012; Tojkander et al., 2012; Maharam et al., 2015; Gupta et al., 2016; Lee and Kumar, 2020). Structurally, SFs are composed of consecutive, sarcomere-like contractile units (CUs). In each CU, 10 to 30 actin filaments are bundled by actin crosslinking proteins including myosin II, α-actinin, and filamin (Cramer et al., 1997; Pellegrin and Mellor, 2007; Kemp and Brieher, 2018). Myosin II converts the energy of ATP hydrolysis into contractile force (Vale and Milligan, 2000; Kasza and Zallen, 2011). This force is dynamically transmitted along the continuum of SF to its endpoints (Hayakawa et al., 2008). At the termini of SFs, FAs connect SFs with the extracellular matrix (ECM). The FA comprises approximately 500 kinds of proteins including integrin, talin, and vinculin that serve as primary proteins mediating the connection between the ECM and SF (Sastry and Burridge, 2000; Kuo et al., 2011; Hirata et al., 2014; Burridge and Guilluy, 2016). FAs attach cells to the ECM and act as a hub to transduce extracellular mechanical cues such as rigidity and topography of ECM to intracellular signals (Humphries et al., 2009; Kanchanawong et al., 2010).

Recent studies highlight cellular responsiveness to ECM substrate stiffness. A well-known cellular response to substrate rigidity is “durotaxis,” where cells seeded on ECMs with varying stiffness exhibit a preference for migrating toward more rigid regions (Lo et al., 2000; Isenberg et al., 2009). Moreover, the stiffness of the ECM also influences cell differentiation. For instance, on softer substrates (<1 kPa), mesenchymal stem cells manifest neuron-like characteristics. In contrast, on stiffer substrates (>30 kPa), they differentiate into osteoblast-like cells (Discher et al., 2005; Engler et al., 2006). Exertion of myosin-generated contractile force to FAs underpins the rigidity-sensing mechanism. Recent studies emphasize the significance of SF mechanical properties in modulating cellular architecture and mechanosensation by altering the efficacy of the contractile force transmission along SFs to FAs (Ehrlicher et al., 2015; Doss et al., 2020; Katsuta et al., 2023). This mini-review aims to illuminate the mechanisms of myosin-generated force transmission along SF and its effects on cell functions such as FA-mediated cell adhesion, ECM rigidity sensing, and cell migration.

## Actin crosslinkers modulate mechanical properties of SFs and the efficiency of contractile force transmission along SFs

**FIGURE 1:**
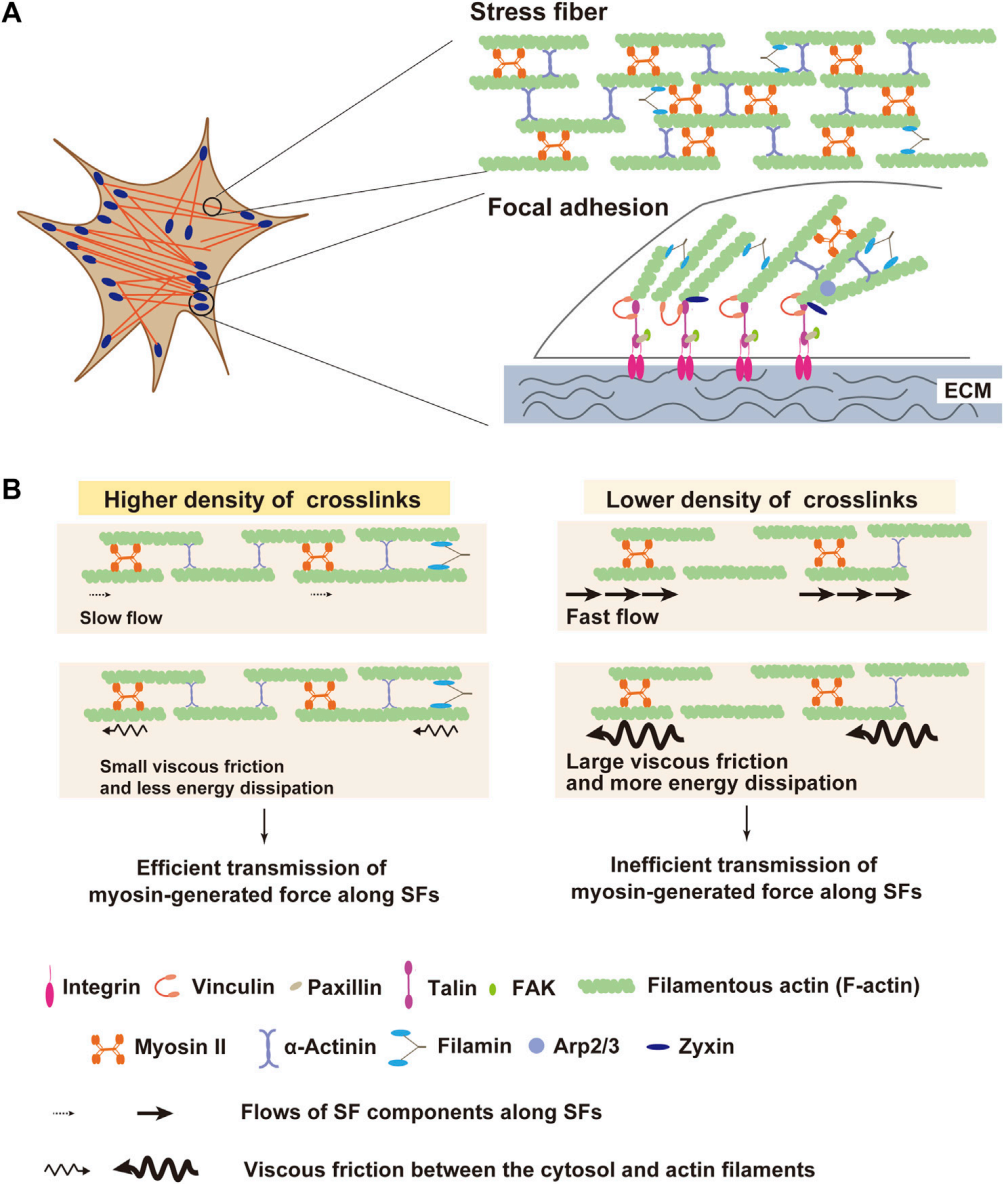
Structures of stress fiber (SF) and focal adhesion (SF) as a machinery of force transmission to extracellular matrix (ECM). **(A)** Schematic diagram of SF and FA. SFs are composed of filamentous actin (F-actin), myosin II, and crosslinking proteins including α-actinin and filamin. At the termini of SFs, FAs connect them with extracellular matrix (ECM). FAs are comprised of ∼500 proteins including integrin, vinculin, talin, and zyxin. Contractile force transmits through SFs to FAs, which enables adherent cells to pull ECM. **(B)** Schematic illustration of the influence of the actin crosslinker density on SF mechanics. Actin crosslinking proteins solidify SFs by reducing the mobility of SF components along SFs. When crosslinkers are sparsely distributed, SFs become fluidized by the increased mobility of SF components. In this situation, myosin-generated energy experiences a large dissipation due to elevated viscous friction between SF and surrounding cytosol, leading to inefficient force transmission along SFs.

In adherent cells, SFs and FAs serve as the primary machinery for anchoring cells to the ECM (Figure 1A). The SF is composed of actin filaments, non-muscle myosin II (NMMII) and actin-crosslinking proteins. SFs play a crucial role in generating myosin-based contractile force and in transmitting generated force to FAs. From the mechanical point of view, the SF acts not just as a passive elastic string but also as an active viscoelastic matter (Kumar et al., 2006; Tanner et al., 2010; Oakes et al., 2017). Subcellular laser ablation has been employed to understand the mechanical properties of SFs *in situ*, where each SF is severed and its viscoelasticity is analyzed from the retraction kinetics of the severed SF. When an SF is incised, it physically retracts throughout its length. The displacement rate of the severed edge was high immediately after severing but slowed down exponentially and eventually became zero at the steady state (Kumar et al., 2006; Wu et al., 2012). This phenomenon was congruent with the Kelvin-Voigt model, characterized as a viscoelastic entity represented by an elastic spring and a viscous dashpot in parallel (Cañadas et al., 2002; Kumar et al., 2006; Tanner et al., 2010).

Actin crosslinking proteins are vital factors that affect mechanical properties of SFs, similar to the case of crosslinkers in synthetic polymer networks (Gardel et al., 2004; Lieleg et al., 2009; Schmoller et al., 2009; Norstrom and Gardel, 2011). For instance, an increase in the affinity of α-actinin for actin has been shown to increase the elasticity of actin filament gels *in vitro* (Wachsstock et al., 1994). Recent studies from our and other groups have shown that several SF-crosslinking proteins including α-actinin, filamin, and non-muscle myosin IIB (NMMIIB) contribute to the elasticity of SFs in living melanoma, osteosarcoma and myoblast cells (Kasza et al., 2009; Weißenbruch et al., 2021; Katsuta et al., 2023). Depletion of all α-actinin isoforms significantly accelerates flows of myosin II and F-actin along SFs, which is driven by the myosin II activity (Katsuta et al., 2023). Thus, α-actinin-mediated crosslinks solidify SFs by reducing the mobility of SF components along SFs. Consistently, atomic force microscopy (AFM) measurements showed that α-actinin depletion drastically decreased the elasticity of SFs (Katsuta et al., 2023). Solidification of the actin cytoskeleton by α-actinin-mediated crosslinks has also been shown *in vitro* using the particle-tracking microrheology method (Ehrlicher et al., 2015), which measures local mechanical properties of the environment by tracking the motions of individual microparticles embedded in the environment (Tseng et al., 2002; Wirtz, 2009).

An increase in the flow speed of the SF components upon α-actinin depletion would elevate viscous resistance between the SF and the surrounding cytosol and, thereby, increase the escape of myosin-generated force from the SF into the cytosol. Thus, it is predicted that SF fluidization in α-actinin-depleted cells may lower the efficiency of transmission of contractile force along the SF (Figure 1B). Consistent with this prediction, α-actinin depletion causes a decrease in traction stress exerted on ECM, even though it does not reduce the myosin activity in SFs (Katsuta et al., 2023). Traction stress exerted on ECM depends on myosin activity (Jorrisch et al., 2013; Chang and Kumar, 2015), and forced activation of myosin with the non-specific phosphatase inhibitor calyculin A increases the traction stress (Jerrell and Parekh, 2014; Katsuta et al., 2023). However, in α-actinin-depleted cells, calyculin A treatment did not increase traction stress (Katsuta et al., 2023), suggesting that elevated myosin II force failed to be transmitted efficiently. Since calyculin A treatment of α-actinin-depleted cells increased the flow speed of SF components, elevated viscous friction between the flowing SF components and the cytosol in calyculin-A-treated cells might impair the calyculin-A-induced increase in traction stress. To our knowledge, no study directly measured viscous resistance in living cells. In general, viscous resistance is proportional to the mobility of the interface in a Newtonian fluid. Given that the cytosol is a Newtonian fluid, when the relationship between the distance from SFs and the displacement rate of the microparticles embedded in the cytosol is measured, it is predicted that the rate gets smaller in a distance-dependent manner. Here, the gradient of the rate to the distance is proportional to the viscous resistance between the SF and the cytosol. This hypothesis awaits future studies.

α-Actinin is accumulated not only along SFs but also at FAs, wherein it binds to both actin and integrin (Roca-Cusachs et al., 2013). This raises another possibility that lowered traction stress in α-actinin-depleted cells might be caused by less efficient force transmission at SF-FA junctions. However, depletion of filamin, another actin crosslinking protein, also lowers traction stress exertion to ECM at FAs without affecting non-muscle myosin II activity (Esue et al., 2009; Kasza et al., 2009; Thomas et al., 2015), suggesting that reduction in expression of actin crosslinkers decreases traction stress by causing dissipation of transmitting force along SFs rather than by disconnecting the SF-FA linkage. This notion is also supported by the fact that the α-actinin 4 mutant with higher binding affinity for F-actin leads to an increase in traction stress (Ehrlicher et al., 2015). This elevation of traction stress may be derived from the attenuation of the flow of actin filaments along SFs in the α-actinin 4 mutant-expressing cells.

Contrary to the results described above, some studies have shown that depletion of α-actinin 4 increases traction stress in fibroblasts (Roca-Cusachs et al., 2013; Doss et al., 2020). One possible basis for the inconsistency is that α-actinin 4 depletion may increase the density of myosin II binding to actin filaments perhaps due to an increase of myosin binding sites along each filament in fibroblasts (Shao et al., 2010a), which possibly elevates contractile force generation. As another possibility, a large difference in the actin binding affinity of α-actinin isoforms may be involved in the inconsistency. α-Actinin 1 and 4 form a heterodimer which is an abundant form of α-actinin dimers in many human cell lines (Foley and Young, 2013). Therefore, depletion of α-actinin 4 is likely to increase the amount of α-actinin 1 homodimers. Since the affinity of α-actinin 1 for actin is about 100 times higher than that of α-actinin 4 (Goldmann and Isenberg, 1993; Weins et al., 2007; Ferrer et al., 2008; Thomas and Robinson, 2017), the shift from α-actinin 1/α-actinin 4 heterodimers to α-actinin 1 homodimers would elevate the stability of actin crosslinks, thereby solidifying SFs and increasing the efficiency of force transmission along SFs. Nevertheless, depletion of α-actinin 1 in α-actinin-4-KD cells, which would result in a decrease of stable actin crosslinks, reduces traction stress (Doss et al., 2020), again suggesting the importance of α-actinin crosslinks in the myosin-generated contractile force transmission. Taken together, it is suggested that actin crosslinkers fine-tune force transmission along SFs by averting fluidization of SFs. Alterations in force transmission would modulate the balance between myosin-based force generation and the magnitude of traction stress exerted on ECM.

Cellular tensegrity is a concept for the intracellular cytoskeletal integrity that the cytoskeleton in a living cell is stabilized by tensile prestress and maintained through a complementary force balance between contractile actomyosin fibers and compression-bearing elements (e.g., nucleus and microtubule) (Ingber, 2003; Ingber et al., 2014). Thus, modulation of SF mechanics by actin crosslinker proteins potentially affects the shape, distribution, and dynamics of the nucleus and microtubules in living cells. Transmission of tensile force to the nucleus is mainly through a class of SFs called perinuclear actin cap that are associated with the apical surface of the nucleus (Khatau et al., 2009). α-Actinin is enriched in the perinuclear actin cap (Shiu et al., 2018), and depletion of α-actinin may reduce force transmission along the perinuclear actin cap to the nucleus. Indeed, α-actinin depletion causes a change in the shape and the position of the nucleus in a cell and disrupts the symmetry of the actomyosin network (Shao et al., 2010a; Maninová and Vomastek, 2016; Senger et al., 2019). Recently, it has been revealed that nuclear deformation by SFs causes opening of nuclear pores, which induces nuclear localization of Yes-associated protein (YAP) (Elosegui-Artola et al., 2017; Panciera et al., 2017; Shiu et al., 2018; Lee et al., 2019; Koushki et al., 2023), a nucleo-cytoplasm-shuttling transcriptional coactivator that promotes gene expression involved in cell proliferation and differentiation (Zhao et al., 2010; Dupont et al., 2011; Pavel et al., 2018). Therefore, actin crosslinker-modulated mechanics of SFs may play a crucial role in the mechanical regulation of YAP-mediated cell proliferation and differentiation, which should be further tested in future studies.

## FA maturation is altered by mechanical properties of SFs

FAs are composed of over 500 kinds of proteins that include adhesion molecules, scaffold proteins, and assorted enzymes (Kuo et al., 2011), and connect SFs to ECM (Figure 1A). FA maturation by protein accumulation depends largely on intracellular and extracellular mechanical conditions, such as myosin contractile force and ECM rigidity (Bremer et al., 1991; Galbraith et al., 2002; Pasapera et al., 2010; Walcott et al., 2011; Rens and Merks, 2020). Force application by optical tweezers to fibronectin-coated beads that attach to the fibroblast surface has shown that fibroblasts respond to the restraining force on beads through local strengthening of the linkage between the beads and the cytoskeleton, allowing stronger force to be exerted on the integrins (Galbraith et al., 2002; Arbore et al., 2019). Conversely, when contractile force generation by myosin II is inhibited with blebbistatin in fibroblasts, compositional maturation of FA is perturbed (Hirata et al., 2008; Pasapera et al., 2010; Oakes et al., 2012). These results suggest that force applied to the FA is essential for FA maturation. As discussed in the above section, the mechanical property of SFs has a large impact on the contractile force transmission along SFs and force exertion to FAs and ECM. Hence, accumulation of FA components might also be modulated by the mechanical properties of SFs. Indeed, studies have shown that depletion of α-actinin, which lowers the efficiency of force transmission along SFs by fluidizing SFs, inhibits FA elongation and maturation in fibroblasts, although nascent FA formation is not affected (Choi et al., 2008; Roca-Cusachs et al., 2013). Moreover, filamin-mediated crosslinks of actin filaments, which make actin filament network more solid-like (Tseng et al., 2004), also increase traction force exertion and contribute to the accumulation of FA proteins in melanoma cells and fibroblasts (Kasza et al., 2009; Lynch et al., 2011).

The FA protein zyxin localizes to matured FAs, but not to nascent adhesions, dependently on tension at FAs, and promotes actin polymerization at FAs (Lele et al., 2006; Hirata et al., 2008; Nguyen et al., 2010; Legerstee et al., 2019). Upon depletion of α-actinin, zyxin is delocalized from FAs in osteosarcoma cells and myoblasts (Feng et al., 2013; Katsuta et al., 2023). Although zyxin could directly bind to α-actinin, a mutant form of zyxin with a deletion of the α-actinin-binding site still localized to FAs (Nix et al., 2001), suggesting that zyxin localization at FAs was not dependent on its direct binding to α-actinin. Interestingly, localization of all the proteins at matured FAs is not affected by the depletion of actin crosslinkers. Vinculin, one of common FA proteins, binds to another FA protein talin in a “force-dependent” manner through the force-induced exposure of vinculin binding sites in a talin molecule (Papagrigoriou et al., 2004; Del Rio et al., 2009; Hirata et al., 2014; Yao et al., 2014). However, multiple studies have shown that localization of vinculin and talin to FAs is independent of the expression level of α-actinin in osteosarcoma cells, myoblasts and keratinocytes (Feng et al., 2013; Hamill et al., 2015; Katsuta et al., 2023). As a potential basis underlying differential effects of α-actinin expression on FA localization of zyxin and vinculin, the force level required for FA localization may be different between zyxin and vinculin. Accumulation of vinculin to FAs was observed at the force larger than 2–3 nN per FA, whilst 10–30 nN per FA was required for zyxin accumulation at FAs (Balaban et al., 2001; Uemura et al., 2011). Although traction force at single FAs is 5–50 nN, which largely differs between FAs, cell types, and rigidity and topography of the extracellular substrate (Trichet et al., 2012; Zhou et al., 2017; Eckert et al., 2021), depletion of α-actinin-mediated crosslinks lowers traction stress by 30%–60% (Katsuta et al., 2023). Thus, α-actinin depletion may lower traction force at FAs to the extent that inhibits zyxin accumulation but is still larger than that required for vinculin accumulation at FAs. Taken together, the actin crosslinker-regulated mechanical property of SFs may be one of the key factors that affect FA maturation by modulating the transmission of contractile force along SFs to FAs.

Interestingly, when α-actinin is depleted, zyxin is not only delocalized from FAs but also accumulated along the entire length of SFs in osteosarcoma cells and myoblasts (Feng et al., 2013; Katsuta et al., 2023). Previous studies have shown that LIM domain proteins including zyxin directly bind to actin filaments in fibroblasts when filaments are tensed by myosin II activity (Smith et al., 2010;Smith et al., 2014). One possible mechanism of zyxin recruitment to SFs with sparse actin crosslinks is as follows; when actin filaments are sparsely crosslinked in an SF, only a small population of actin filaments would be involved in transmitting myosin force along the SF at each time point and sustain a larger tensile force than those in SFs with a high crosslink density. These highly tensed actin filaments could provide binding sites of zyxin in α-actinin-depleted SFs. Consistently, stretching of SFs with a normal α-actinin level also causes zyxin accumulation along SFs in fibroblasts (Yoshigi et al., 2005). Zyxin accumulated along SFs promotes actin polymerization with the aid of Ena/VASP proteins and, thereby, structurally reinforces SFs that are subjected to mechanical challenges such as excess extension or depletion of actin crosslinkers (Yoshigi et al., 2005; Smith et al., 2010; Katsuta et al., 2023).

## Possible roles of SF mechanics in ECM rigidity sensing and cell migration

**FIGURE 2:**
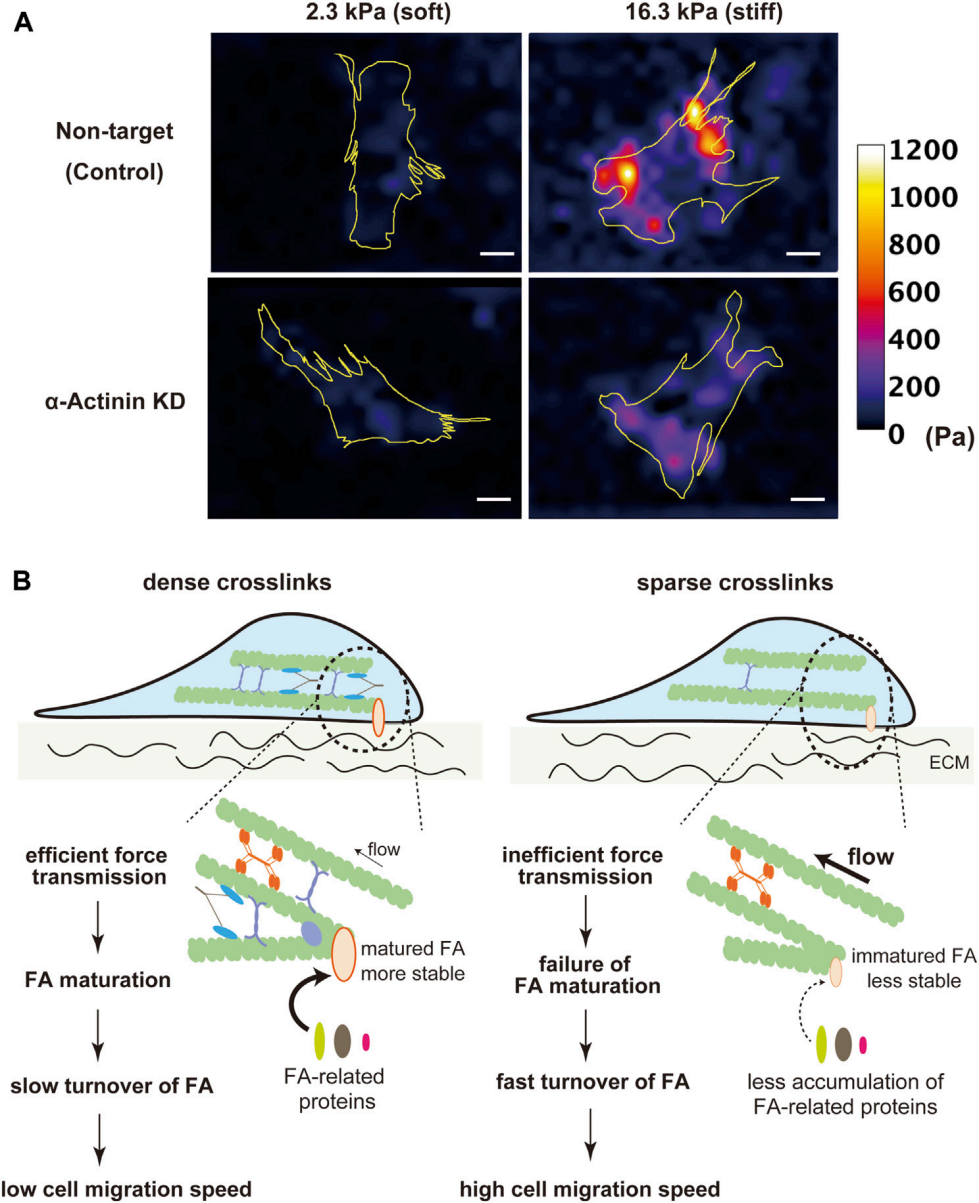
Contribution of actin crosslinking proteins to ECM rigidity sensing. **(A)** Traction stress images of α-actinin KD and non-target (control) C2C12 myoblast cells on fibronectin-coated 2.3 kPa and 16.3 kPa polyacrylamide gel substrates. Non-target cells on stiffer substrates exerted larger traction stress than on softer substrates. The rigidity-dependent increase in traction stress was attenuated in α-actinin KD cells. Yellow lines indicate outlines of cells. The heatmap scale of traction stress is common between non-target and α-actinin KD cells. Scale bars, 20 μm. **(B)** Schematic diagram of the possible roles of actin crosslinkers in cell migration. Cells with a high density of actin crosslinking proteins transmit contractile force more efficiently to FAs, which stabilizes FAs, attenuates FA turnover, and, thereby, lowers the cell migration speed.

One of the essential roles of the SF-FA system is to sense the rigidity of ECM. At FAs, cells sense the rigidity of ECM by pulling it with myosin-generated contractile force (Lohner et al., 2019; Doss et al., 2020). As SF mechanics mediated by actin crosslinking proteins largely affect traction force exertion through modulation of force transmission along SFs, actin crosslinking proteins may be involved in cellular sensing of the substrate rigidity. Myosin II is more activated in fibroblasts on stiffer substrates, which leads to exerting larger traction stress on the substrates (Schäfer and Radmacher, 2005; Frey et al., 2006; Fouchard et al., 2011). However, melanoma and myoblast cells deficient in α-actinin or filamin A exhibit a failure in the substrate rigidity-dependent increase in traction stress (Byfield et al., 2009; Katsuta et al., 2023) (Figure 2A). This suggests a possibility that a decrease in actin crosslinks hampers rigidity sensing at FAs due to lowered efficiency of force transmission to FAs. At the leading edge of a fibroblast, a single-sarcomere-like complex called contractile unit (CU) has been reported as a minimal machinery for rigidity sensing (Ghassemi et al., 2012; Wolfenson et al., 2015; Meacci et al., 2016). Similar to SFs, CUs are composed of actin and myosin II filaments, tropomyosin, and α-actinin (Ghassemi et al., 2012; Wolfenson et al., 2015; Meacci et al., 2016). CUs link the adjacent integrin clusters separated by 1–2 μm, and pull on these integrin clusters to locally deform ECM (Ghassemi et al., 2012). Recruitment of α-actinin to CUs has been reported to be an essential step for CUs to exert a certain level of force required for sensing ECM rigidity (Wolfenson et al., 2015; Meacci et al., 2016). It is conceivable that actin crosslinking proteins avert slippage of actin and myosin II filaments in both SFs of multiple sarcomere units in series and CUs of single-sarcomere units.

Cell migration is another cellular activity that is regulated by intracellular and extracellular mechanical conditions. During cell migration, traction force exerted from FAs to ECM drives forward translocation of the cell body (Fournier et al., 2010; Yamaguchi and Knaut, 2022). On the other hand, even though assembly/disassembly dynamics of FAs are required for continuous cell migration, the tensile force acting on FAs stabilizes them and hampers their disassembly (Trichet et al., 2012; Zhou et al., 2017). Therefore, the velocity of cell migration is determined by a balance between these opposing effects of myosin-based force exerted on FAs. Since actin crosslinkers largely affect force transmission along SFs to FAs, the cell migration velocity is potentially regulated by the expression level of actin crosslinkers. Indeed, previous studies have shown an inverse relationship between the expression levels of actin crosslinking proteins, such as α-actinin, myosin IIB, or filamin, and the velocity of fibroblast migration (Glück and Ben-Ze’ev, 1994; Lo et al., 2003; Ketebo et al., 2021). With a low density of actin crosslinking proteins, smaller force exerting on FAs makes FAs less stable, leading to an increase in the speed of cell migration with a higher FA turnover rate (Figure 2B).

ECM stiffness also affects cell migration, mainly through alteration of FA stability (Lange and Fabry, 2013; Hadden et al., 2017). One outstanding example is durotaxis, in which cells migrate from softer to stiffer substrates (Lo et al., 2000; Isenberg et al., 2009). A proposed mechanism for durotaxis is that cellular protrusions on stiffer regions exert larger force to FAs, which makes FAs more stable, leading to a movement of the cell body toward stiffer regions (Plotnikov et al., 2012; Shellard and Mayor, 2021). Since actin crosslinkers can modulate rigidity sensing and cell migration, it is possible that actin crosslinking proteins affect durotaxis. Indeed, a small reduction of MYHIIB expression significantly diminished durotaxis (Raab et al., 2012), which supports the possibility that actin crosslinkers contribute to rigidity-dependent migration.

It has been well established that cancer cells plated on stiff substrates show more aggressive phenotypes than those on soft substrates (Paszek et al., 2005; McKenzie et al., 2018; DuChez et al., 2019; Espina et al., 2022). Actin crosslinking proteins, α-actinin, myosin II, and fascin, are highly upregulated in multiple human cancers, where they promote invasive cell behaviors and correlate with poor patient prognosis (Vignjevic et al., 2007; Jayo and Parsons, 2010; Jansen et al., 2011; Ouderkirk and Krendel, 2014; Schoumacher et al., 2014; Jayo et al., 2016; Chen et al., 2021). While the detailed mechanism of how the upregulated expression of actin crosslinkers alters cancer cell behaviors remains to be elucidated, it is possible that by increasing efficiency of force transmission to FAs, actin crosslinkers may enable exertion of large traction stress even on soft substrates to promote maturation of FAs. Force-induced maturation of FAs would lead to activation of FA-mediated signaling including the focal adhesion kinase (FAK) pathway that is essential for enhancing proliferation and invasion of cancer cells (Chan et al., 2009; Shibue and Weinberg, 2009; Pasapera et al., 2010).

Whilst discussion above is mainly based on results obtained using cells cultured on 2D substrates, recent studies have shown that cell migration modes are different in between 2D and 3D environments. Fibroblasts, smooth muscle cells and some cancer cells in 3D ECM exhibit mesenchymal migration which is characterized by an elongated cell shape with long membrane protrusions, strong adhesion to surrounding ECM, and proteolytic degradation of the ECM (Friedl and Weigelin, 2008). This type of cell migration depends on actomyosin contractility-induced stabilization of cell adhesion to ECM (Doyle et al., 2015). Filamin A is required for mesenchymal migration of macrophages through the formation of cell adhesions to 3D ECM (Guiet et al., 2012), which is consistent with our notion in 2D that sparse crosslinks of actin filaments destabilize cell adhesion due to impaired contractile force transmission to adhesion sites. Impairments in FA maturation and cell migration in 3D environments are also observed upon filamin B depletion in A549 lung carcinoma cells and HT1080 fibrosarcoma cells (Iguchi et al., 2015). However, compared with the case of cell migration on the 2D surface, knowledge about the role of actin crosslinking proteins in cell migration in 3D environments is limited and needs to be revealed in future studies.

## Actin-crosslinking proteins modulate cellular responses to externally applied mechanical stimuli

In living tissues, cells are exposed to various kinds of mechanical stresses including stretch and shear stress, and these external mechanical stimuli alter cell behaviors (Janmey and Weitz, 2004). For instance, wound healing is accelerated by uniaxial cyclic stretch (Skutek et al., 2001; Huang et al., 2013; Kawai et al., 2023), and laminar shear stress applied to vascular endothelial cells relaxes vascular smooth muscle by promoting nitric oxide production (Harrison et al., 2006; Ando and Yamamoto, 2010). External forces act on or are transmitted to the SF-FA complex, which causes cellular responses (Paul et al., 2008; Colombelli et al., 2009).

Sustained stretch of the extracellular substrate induces rapid FA growth and tyrosine phosphorylation of FAK at FAs (Wang et al., 2001; Chen et al., 2013). Actin crosslinking by α-actinin or filamin causes stiffening of actin networks in response to the stretch (Xu et al., 2000; Schmoller et al., 2010), which would enhance stretch-induced development of tension at FAs that connect between the actin cytoskeleton and the extracellular substrate (Kumar et al., 2019). Thus, actin crosslinkers may facilitate mechanotransduction at FAs in response to stretching of the extracellular substrate.

When cells are subjected to uniaxial cyclic stretching, SFs oriented in parallel to stretch axis are disassembled (Dartsch and Betz, 1989; Roshanzadeh et al., 2020). This process relies on severing of unloaded actin filaments by cofilin (Hayakawa et al., 2011). If an SF is a pure elastic object, tensile stress is repeatedly increased in the cyclically stretched SF in phase with the stretch cycle. By contrast, when a totally viscous object is subjected to cyclic stretching, tensile and compressive stresses are developed alternately in the object (Stamenović, 2008; Hirata and Sokabe, 2022). Since actin crosslinkers modulate the viscoelastic property of SFs, the difference in the crosslinker density in SFs should have a large impact on mechanical stress development in SFs in response to cyclic stretching. Especially, in less elastic SFs with a low density of actin crosslinkers, cyclic stretching would cause development of a significant compressive stress in SFs, which is likely to decrease myosin-based pretension in actin filaments within the SFs, thereby promoting cofilin-dependent severing of the actin filaments and disassembly of the SFs. Such enhanced disassembly of less-crosslinked SFs oriented in parallel to the axis of uniaxial cyclic stretching may facilitate the reorientation of SFs and cells in response to the stretching; both SFs and cells become aligned perpendicular to the stretch axis (Zielinski et al., 2018; Kumar et al., 2019).

Although interactions between filamentous actin and actin crosslinking proteins can withstand external strains during deformation of actin cytoskeleton through unfolding and conformational transitions of crosslinking proteins (Rivero et al., 1996; D’Addario et al., 2001; Ferrer et al., 2008; Jackson et al., 2008; Hoffman et al., 2012; Razinia et al., 2012; Le et al., 2017), excessive stiffening of SFs with a high density of actin crosslinkers may conversely make cells fragile against mechanical deformation. Podocytes expressing α-actinin 4 mutants with increased binding affinity to F-actin, which induce familial focal segmental glomerular sclerosis (Kaplan et al., 2000), show breakages in their actin cytoskeleton upon periodic 10% uniaxial stretch (Feng et al., 2018; Feng et al., 2020). Such breakage might be caused by reduced plasticity of the actin cytoskeleton with tight crosslinks of actin filaments. Although further studies are required to elucidate how actin crosslinking proteins alter cytoskeleton remodeling in living cells under various mechanical stimuli, it is possible that they largely regulate mechanical response of SFs and FAs. Furthermore, it is notable that conformational changes of actin crosslinking proteins in response to deformation of the actin network may also contribute to cellular mechanotransduction. For example, mechanical strain of the filamin A-crosslinked actin network causes conformational changes of filamin A, altering its interaction with its binding partners including β1 integrin and FilGAP (Ehrlicher et al., 2011).

## Concluding remarks

The complex interplay between SFs, FAs, and ECM is fundamental to diverse cellular functions including migration, proliferation and differentiation. In this review, we have focused on the effects of actin crosslinking proteins, which stabilize SFs and play pivotal roles in FA maturation, substrate rigidity sensing, cell migration, and mechanotransduction. Mutations in these crosslinkers have been revealed to be associated with a diverse spectrum of pathologies. For instance, mutations in α-actinin isoforms that upregulate or downregulate stability of actin crosslinks have been linked to several human diseases including autosomal-dominant congenital macrothrombocytopenia (Kanhai et al., 2018; O’Sullivan et al., 2021), dilated or hypertrophic cardiomyopathy (Lindholm et al., 2021), familial form of FSGS (Kaplan et al., 2000; Yang and Glass, 2008), and immunological diseases (Kikonomou et al., 2011). Mutations in filamin A also induce a wide spectrum of diseases including skeletal dysplasia, neuronal migration abnormality, cardiovascular malformation, intellectual disability, and intestinal obstruction (Robertson et al., 2003; Robertson, 2005; Eltahir et al., 2016; Sasaki et al., 2019). An *in-vivo* study has shown that depletion of filamin A in vascular smooth muscle cells induces lower blood pressure due to aortic dilation and increases in atrial compliance (Retailleau et al., 2016). Furthermore, multiple proteomic studies have implicated fascin contribution to several neurological diseases, such as seizure and Alzheimer disease (Spencer et al., 2011; Lamb and Tootle, 2020). While detailed mechanisms of how these protein mutations cause disease pathogenesis are still under active investigation, alteration of the mechanical properties of SFs can be involved in disease manifestation.

The actin binding affinity of actin crosslinkers including α-actinin, filamin, and plastin can be modulated by intracellular Ca^2+^ levels (Burridge and Feramisco, 1981; Glenney et al., 1981; Nakamura et al., 2005; Kasza et al., 2009; Foley and Young, 2014; Drmota Prebil et al., 2016; Lehne and Bogdan, 2023). Ca^2+^ binding to EF-hands of these actin-binding proteins decreases their affinity for actin binding and elasticity of the actin network *in vitro* (Pinotsis et al., 2020). Consistent with this, a decrease in the intracellular Ca^2+^ concentration ([Ca^2+^]_i_) causes an increase in cytoskeletal stiffness, suggesting that intracellular Ca^2+^ modulates actin crosslinker-mediated mechanical properties of the actin cytoskeleton (Brundage et al., 1991; Wei et al., 2012). Accordingly, the transmission of myosin-generated force in the actin cytoskeleton to FAs might be increased under the low [Ca^2+^]_i_ condition, which was supported by the finding that FAs were stabilized and cell motility was hampered under such condition (Wei et al., 2012). [Ca^2+^]_i_ is upregulated by various extracellular stimuli. For example, a range of ligands for G-protein-coupled receptors (GPCRs) induces IP_3_-mediated Ca^2+^ release from intracellular Ca^2+^ stores such as endoplasmic reticulum (Lock et al., 2019; Wang et al., 2019; Woll and Van Petegem, 2021), and mechanical stresses (e.g., cyclic stretch and shear stress) cause Ca^2+^ influxes via activation of mechanosensitive channels (Clapham, 2007; Murata et al., 2014; Sato et al., 2015). Substrate stiffness also regulates the magnitude and the frequency of [Ca^2+^]_i_ increases in mesenchymal stem cells (Kim et al., 2009). Binding of phosphatidylinositol 4,5-bisphosphate (PIP_2_) to actin crosslinker proteins also modulates their affinity for actin (Greenwood et al., 2000; Izaguirre et al., 2001; Fraley et al., 2003; Shao et al., 2010b; Foley and Young, 2014). The intracellular level of PIP_2_ is regulated by various ligands for GPCRs or receptor tyrosine kinases (e.g., PDGF receptor) through activation of phospholipase C (PLC), phosphoinositide 3-kinase (PI3K), or phosphatase and tensin homologue deleted on chromosome 10 (PTEN) (Balla, 2013; Cocco et al., 2015; Liu et al., 2018; Harraz et al., 2020; He et al., 2021). Taken together, it is conceivable that actin crosslinking and SF mechanics are potentially modulated by multiple physical and chemical factors in the extracellular environments.

Despite substantial attempts to reveal SF-FA mechanics and its role in cell behaviors, much remains elusive. First, from both molecular and biophysical perspectives, we need more observations to quantitatively understand how actin crosslinking proteins alter mechanical properties of SFs, FA maturation and rigidity sensing. Second, although the affinity of actin crosslinker proteins for actin is affected by Ca^2+^ and PIP_2_, when and where cells utilize these regulation systems remain to be solved. Furthermore, it is an open question how malignant cells alter the rigidity sensing systems to facilitate their invasiveness and proliferation. Further studies focusing on these points will significantly enhance our understanding of how actin crosslinker-dependent modulation of the SF-FA machinery contributes to regulations of cellular functions.
